# Do Cannabinoids Confer Neuroprotection Against Epilepsy? An Overview

**DOI:** 10.2174/1874205X01711010061

**Published:** 2017-12-18

**Authors:** Anna Capasso

**Affiliations:** Department of Pharmacy, University of Salerno, *via* Giovanni Paolo II, 84084, Fisciano, Italy

**Keywords:** Antiepileptic, Cannabinoids, Epilepsy, Neuroprotection, Cannabis sativa

## Abstract

**Objective:**

Cannabinoid-based medications provide not only relief for specific symptoms, but also arrest or delay of disease progression in patients with pain, multiple sclerosis, and other conditions. Although they also seem to hold potential as anticonvulsant agents, evidence of their efficacy in epilepsy is supported by several evidences.

**Method:**

The data reviewed herein lend support to the notion that the endocannabinoid signalling system plays a key modulation role in the activities subserved by the hippocampus, which is directly or indirectly affected in epilepsy patients.

**Conclusion:**

The notion is supported by a variety of anatomical, electrophysiological, biochemical and pharmacological findings. These data suggest the need for developing novel treatments using compounds that selectively target individual elements of the endocannabinoid signalling system.

## 1 INTRODUCTION

*Cannabis sativa* is a well-known recreational drug [1], whose millenarian medicinal use has only recently been revisited [2]. Indeed, as far back as 2600 BC, the Chinese emperor Huang Ti recommended using it to relieve cramps and rheumatic and menstrual pain [3, 4].

The endocannabinoid system (ECS) is a novel endogenous signalling system involved in a variety of functions both in physiological and pathological conditions. It consists of cannabinoid receptors type 1 (CB1) [5] and 2 (CB2) [6], their endogenous fatty-acid ligands (endogenous cannabinoids or endocannabinoids, ECs), and the proteins responsible for EC biosynthesis and degradation. CB1 receptor is chiefly expressed in the central nervous system (CNS) and, to a lesser extent, in peripheral nerve terminals and at various non-neuronal sites such as testis, uterus, eye, vascular endothelium, spleen, and adipocytes [7-10], whereas CB2 receptor is nearly exclusively expressed in the immune system. Although the existence of other cannabinoid receptors has been suggested by pharmacological studies, they have not yet been cloned [11].

The two receptors exhibit only 44% and 68% overall identity in transmembrane domains. They are mainly coupled to G proteins, most often of G_i/o_ type, inhibiting adenylate cyclase activity and stimulating mitogen-activated protein kinases through α subunit. Coupling to ion channels, resulting in the inhibition of Ca^2^+ influx *via* N-type calcium channels, has also been described [12]. In addition, CB1 receptor is involved in the activation of PI-3-kinase and phospholipase C (through the G protein βγ subunits), whereas CB2 receptor plays a role in inducing sustained activation of ceramide biosynthesis [13].

## 2 ENDOCANNABINOID SYNTHESIS, RELEASE, UPTAKE, AND DEGRADATION

Their highly lipophilic nature entails that ECs, unlike other neurotransmitters, cannot be stored in vesicles, but rather are synthesized on demand. EC signalling is therefore closely regulated by their synthesis, release, uptake, and degradation processes [14]. A variety of stimuli, including membrane depolarization and increased intracellular Ca^2+^ and/or receptor stimulation, are capable of activating the process that through cleavage of membrane phospholipids leads to EC synthesis. Notably, different enzymes participate in the synthesis of the various ECs; as a consequence, different ECs are involved in different physiological and pathological conditions.

In animal tissues, anandamide (or N-arachidonoylethanolamide, AEA) and its congeners, collectively denominated N-acylethanolamines, are mainly formed from their corresponding N-acyl-phosphatidylethanolamine (NAPE) by a phospholipase D (PLD) phosphodiesterase [15]; in turn, N-acylethanolamines are mainly synthesized from membrane phospholipids by enzyme reactions involving first N-acylation of phosphatidylethanolamine by an acyltransferase, and subsequently N-acylethanolamine release from NAPE through the action of a PLD phosphodiesterase [16].

## 3 THE ENDOCANNABINOID SYSTEM

Several ECs have been described to date. AEA was the first to be discovered, in 1992, followed by 2-arachidonoylglycerol (2-AG). Both are arachidonic acid derivatives and can bind to CB1 and CB2 receptors, albeit with different affinity and activation efficacy [8]. Several other bioactive lipid mediators that appear to exert distinct pharmacological effects in vivo through CB1 and/or CB2 receptors, have subsequently been described, including 2-arachidonoyl-glyceryl-ether (noladin ether), o-arachidonoyl-ethanolamine (virodhamine), N- arachidonoyl-dopamine, and possibly oleamide [13, 10, 17, 18]

The biological roles of the ECS have thoroughly been explored [19, 20]. The ECS participates in a large number of functions in physiological conditions. In the CNS, ECs are involved in short and long-term synaptic plasticity including depolarization-induced suppression of excitatory/inhibitory neurotransmission, long-term potentiation/depression, and long-term depression of inhibition [21], participating in the regulation of cognitive functions and emotions in neuronal circuits of the cortex, hippocampus, and amygdala, and in the reinforcement of substances of abuse in the mesolimbic system [22]. In the basal ganglia and cerebellum, strong expression of CB1 receptor and ECs involves effects on movement and posture, for instance through an action on dopaminergic signalling [23]. In addition, neuromodulation exerted on the sensory and autonomic nervous systems affects pain perception [24], cardiovascular [25] and gastrointestinal function [26], mostly *via* CB1 receptor. Through crosstalk with steroid hormones, hypothalamic hormones and peptides, ECs also affect food intake, the pituitary-hypothalamus-adrenal axis and reproduction [27]. CB2 receptor is involved in cellular and, especially, in humoral immune responses, and through them, it may exert effects on (neuro) inflammation and chronic pain [28].

Interestingly, ECs also inhibit tumour cell proliferation; in particular, data obtained from isolated cells suggest that AEA and 2-AG are involved in cell metabolism, differentiation, proliferation and death *via* cannabinoid and non-cannabinoid receptors [29].

In addition to the above physiological functions, where demonstration of their “tonic” nature has been provided only for some of them, EC signalling shows striking tissue-specific changes in pathological conditions [30]. In transient disorders, overexpression of at least one EC in the tissue(s) involved, contributes to restoring normal levels of other endogenous mediators *via* CB1 receptor. This has been observed seen for instance in some areas of the nervous system affected by injury or stressors, such as the hippocampus in an animal model of epilepsy [30], the hypothalamus following food deprivation [31], and the spinal cord in an animal model of multiple sclerosis [32].

Since cannabis-based medicines have been reported to have both anticonvulsant [27-29, 33-35] and proconvulsant effects [36], it is important to establish whether cannabinoids can be harnessed to treat CNS hyperexcitability or rather constitute a potential risk to recreational and medicinal users [37].

Although the association between epilepsy and the EC receptor system has extensively been explored, the complex relationship between brain excitability and the ECS suggests that cannabinoids may have beneficial effects on epilepsy [27-38].

## 4 EPILEPSY AND ANTIEPILEPTIC DRUGS

Epilepsy involves a broad spectrum of disorders that affect about 50 million people in the world [39].

Epileptic seizures are mainly divided into partial seizures - which arise in specific brain regions, especially the temporal lobes and the hippocampus and do not spread to other areas - and generalized seizures - involving the entire forebrain through secondary generalization of partial seizures. Revision of the concepts, terminology, and approaches to the classification of epilepsies and seizures by the Commission on Classification and Terminology of the International League Against Epilepsy (ILAE) has provided a subdivision of seizures into those “originating at some point within, and rapidly engaging bilaterally distributed networks” (generalized) and those “originating in networks limited to one hemisphere”, either “discretely localized or more widely distributed” (focal) [39]. The Commission also simplified the classification of generalized seizures; moreover, since a natural classification for focal seizures has not yet been devised, it recommended that focal seizures be described in relation to their manifestation features (*e.g.*, dyscognitive, focal, motor). The notions of generalized / focal do not apply to electroclinical syndromes [39].

As regards form, epilepsy is chiefly characterized by specificity. Here, too, the ILAE revision has introduced a new terminology, where genetic, structural/metabolic, and unknown disorder have replaced idiopathic, symptomatic, and cryptogenic epilepsies [39]. These categories can be further subdivided according to criteria like natural class (*e.g.* underlying cause, age at onset, associated seizure type) or pragmatic groups (*e.g.* epileptic encephalopathies, self-limited electroclinical syndromes), to organize data about recognized forms and contribute to identify new ones [39].

Current antiepileptic drugs (AEDs) include acetozolamide, carbamazepine, clobazam, clonazepam, ethosuximide, eslicarbazepine acetate, gabapentin, lacosamide, lamotrigine, levetiracetam, oxcarbazepine, perampanel, phenobarbital, phenytoin, pregabalin, primidone, rufinamide, sodium valproate, tiagabine, topiramate, valproate, vigabatrin, and zonisamide [40] (Table **1**).

## 5 EVIDENCE FOR THE EFFECT OF CANNABINOIDS AS ANTIEPILEPTICS

Despite the conflicting data reported on the relationship between cannabinoids and brain excitability [27-38], there is evidence that cannabinoids may be beneficial in treating CNS hyperexcitability [33-35].

In one of the earliest studies (1970), Mechoulam [41] randomized nine patients with treatment refractory temporal lobe epilepsy to receive cannabidiol (CBD) or placebo for 5 weeks in addition to their current AED. At 3 months, two of the four CBD patients were seizure-free, whereas none of the five placebo patients showed improvement. In 1975, Consroe and co-workers [42] described a young man whose epilepsy was refractory to standard AEDs (phenobarbital and phenytoin). He reported that seizures had stopped when he had begun smoking cannabis socially; however, AED withdrawal resulted in their return, despite the persistent use of cannabis. Next, Ng and colleagues reported a reduction in seizure behaviour in a population of 308 epileptic patients who used cannabis compared with a control population of 294 patients [43]. All three studies suggested that cannabis may exert an anticonvulsant effect.

Subsequent trials have confirmed the anti-seizure properties of cannabinoids [44-46].

Cunha and colleagues [44] described a double-blind phase 1 study involving 16 healthy volunteers, of whom eight received 3 mg/kg daily CBD and eight received a placebo for 30 days. In the double-blind phase 2 study, 15 patients with treatment-refractory epilepsy (TRE) were randomly assigned to receive CBD (200-300 mg daily) or a placebo for 4 and a half months in addition to their current AEDs. Neither severe side effects nor toxicity were reported by participants in the phase 1 or phase 2 study; in particular, four of the eight CBD patients were seizure-free throughout the study and three showed partial improvement; only one patient failed to improve. Seven placebo patients experienced no clinical benefit, whereas one showed marked improvement. The authors concluded that CBD has potential as an antiepileptic agent and that it may strengthen the effect of other antiepileptic drugs.

According to other researchers CBD has little or no effect. In a study that reported neither the statistical analysis of data nor the main effects, Trembly and Sherman found no discernible benefit of marijuana on TRE [45]. In a small sample, Ames and Cridland [46] found no differences between CBD and placebo and provided no power calculations.

Adult patients taking marijuana and parents treating their TRE children with CBD-enriched marijuana are described in several reports [47-49]. However, despite some encouraging results, there is no consensus on formulation, dosage, route of administration, or treatment duration. The placebo effect and recall bias may be confounding variables. Finally, two of five institutionalized children refractory to phenobarbital and phenytoin showed complete and considerable improvement, respectively, after tetrahydrocannabinol (THC) administration, whereas the other three did no worse than with their previous treatment [50]. Altogether, safety and efficacy data from randomized, controlled trials (RCTs) are quite limited.

## 6 MECHANISM UNDERPINNING THE ANTIEPILEPTIC ACTION OF CBD

The molecular mechanisms subserving the antiepileptic action of cannabinoids, reported by some studies, are still largely unclear. However, two investigations using conditional knockout mice have demonstrated the critical involvement of forebrain glutamatergic neurons in cannabinoid-mediated protection against seizures and rapidly increased EC levels in response to seizures [51, 52].

Using a rat pilocarpine model of epilepsy, Wallace and colleagues [51] found that cannabinoids reduced seizure behaviour more effectively than phenobarbital and phenytoin, suggesting that they may be administered to patients unresponsive to current AEDs. They also showed that the EC tone modulates seizure termination and duration through CB1 receptor activation. Western blotting and immunohistochemical analysis disclosed a significantly increased expression of CB1 receptor protein throughout the CA regions of the epileptic hippocampus. These findings indicate that the ECS is involved not only in regulating seizure activity, but also in modulating neuroexcitation, suggesting that epilepsy induces CB1 receptor plasticity [51].

In a study where they measured EC levels in mouse hippocampus after kainic acid–induced seizures, Marsicano and co-workers [52] provided further support for the involvement of ECs in protection against seizure activity. The seizures induced a rapid AEA increase, hence activation of CB1 receptor, thus providing protection against acute excitotoxicity and activation of protective intracellular signalling cascades. Such findings, obtained in conditional knockout mice, indicate that healthy principal forebrain neurons are required for neuroprotection. In a study of several models of neuronal damage, van der Stelt and co-workers [53] also documented the involvement of CB1 receptor in protection against epilepsy through mechanisms that include CB1-mediated inhibition of glutamatergic transmission, inhibition of harmful cascade signals, and reduction of Ca^2+^ influx. The EC release detected in presence of neuronal injury may therefore be considered as a protective reaction [52,53].

The latter two studies [52,53] strongly support the notion that ECS stimulation during seizures confers significant neuroprotection against neuronal hyperactivity through reduction of hippocampal pyramidal neuron excitability and activation of intracellular signalling cascades. CB1 receptors found on principal glutamatergic neurons in the forebrain are chiefly responsible for this action.

Evidence for a strong relationship among hippocampal glutamatergic neurons, ECs, and neuroprotection against seizures has also been provided by Monory and colleagues [54]. Using novel conditional mutants lacking CB1 receptor in specific neuronal populations and the KA model of seizures, the authors reported functional and anatomical evidence that CB1 receptors found on hippocampal glutamatergic neurons are crucially involved in protection against acute excitotoxic seizures.

Even though the molecular mechanisms underpinning the anticonvulsant action of cannabinoids are still unclear, release of presynaptic glutamate (Glu) after CB1 receptor activation is believed to play a critical role. In fact, CB1 receptor activation:

- reduces Ca^2+^ influx [55], reducing Ca^2+^-dependent Glu release. Since Glu is the primary CNS excitatory neurotransmitter, and epilepsy is related to excess glutamatergic transmission, cannabinoid-induced reduction in Glu release induces an anticonvulsant effect [56];
- enhances presynaptic A-type [57] and G-protein-coupled inward rectifying K^+^ channel conductance [58]. N- and Q-type calcium current inhibition reduces Glu release and synaptic transmission, besides suppressing other calcium-dependent processes. K^+ ^channel activation reduces neuronal excitability through stabilization both of membrane potentials and of other factors involved in reduction of epileptiform discharge [58];
- reduces hippocampal GABAergic function through retrograde signalling, thus modulating synaptic activity. ECs are considered as retrograde mediators of depolarization-induced suppression of inhibition (DSI) [59]. After depolarization, reduction of inhibitory GABA-mediated neurotransmission due to EC release from the depolarized neuron and EC diffusion to nearby neurons result in binding and activation of CB1 receptors, which reduce GABA release through a presynaptic action. Although the reduced GABAergic tone is unlikely to mediate the anticonvulsant effect of cannabinoids [59], Cohen and colleagues [60] have demonstrated that GABA, though normally an inhibitory neurotransmitter, can induce a depolarizing action that is capable of synchronizing abnormal bursting in slice preparations of human epileptic temporal lobe. The authors suggested that if the phenomenon were documented in animals with pilocarpine-induced epilepsy, the cannabinoid-mediated decrease in GABAergic tone would likely exert an anticonvulsant effect (Table **2**) summarizes the experimental and human data regarding the role of cannabidiol in different model of epilepsy.

## 7 SAFETY OF CANNABINOIDS IN EPILEPSY TREATMENT

Although several studies have documented the safety of CBD as an antiepileptic agent, clinical research into the safety and efficacy of cannabinoids has been hampered by the lack of pure, pharmacologically active compounds and by legal constraints [47-50, 69]. According to a recent review [70], the limited available data on the efficacy of cannabinoids as monotherapy or co-treatment for epilepsy prevent drawing reliable conclusions. Only four (blinded and non-blinded) studies, conducted from 1978 to 1990, were found to meet RCT criteria. However, they all involved small samples (48 patients in total) and were too short (4 weeks to 18 months) to assess the safety of long-term CBD treatment. Reported outcomes included reductions in seizure frequency and freedom from seizures [70].

A systematic review of the efficacy and safety of medical marijuana in selected neurological disorders, carried out by the American Academy of Neurology in 2014, found that: i) the efficacy of oral cannabinoids in epilepsy is unknown, ii) the risk-benefit ratio of medical marijuana requires careful assessment, and iii) the effectiveness of medical marijuana compared with other epilepsy treatments is unknown [71]. However, three studies exploring the efficacy and safety of a purified 98% oil-based extract of CBD [72, 73] or of plant-derived CBD [74], which were conducted preliminary to an RCT, reported encouraging data on its ability to control seizures.

The first study [72] enrolled 23 TRE patients with an average age of 10 years, most of whom suffered from Dravet Syndrome (DS), a rare and catastrophic childhood TRE. After assessment of baseline seizure frequency and type and establishment of AED regimens over a period of 4 weeks, patients received 5 mg/kg/day of the purified 98% oil-based CBD extract, which had a known and constant composition, in addition to their current AEDs. The daily dose was progressively increased to 25 mg/kg/day unless intolerance was reported. After three months, 39% of patients achieved a seizure reduction greater than 50% (median 32%); in particular, 3/9 DS patients and 1/14 patients with other forms of epilepsy became seizure-free. Adverse effects were largely mild or moderate and included sleepiness, fatigue, increase in AED dosage, reduced or increased appetite, weight gain or loss, and diarrhoea. These data are encouraging, mainly because they involved a group of children and young adults with highly TRE [72].

The second study [73] assessed the interactions between the CBD extract and current AED regimen in 33 epileptic patients whose average age was 10 years. Patients' current treatment included on average three different AEDs: clobazam (54.5% of patients), valproate (36.4%), levetiracetam (30.3%), felbamate (21.2%), lamotrigine (18.2%), and zonisamide (18.2%). After establishing the baseline dosage of each AED regimen, the CBD extract was added at a starting dose of 5 mg/kg/day; the dose was then raised weekly by 5 mg/kg/day until 25 mg/kg/day. The extract was seen to induce changes in the serum concentrations of some AEDs. For instance, increased clobazam levels, found in a patient subset, were thought to be causing sedation and led to a dose adjustment, suggesting that CBD may affect the main clobazam metabolic pathways. These clinical findings support experimental evidence that CBD can affect the metabolism of some common AEDs, even though these effects are not seen in all patients. Clearly, further work is needed to understand the complex interactions between CBD and AEDs; in the mean time, close monitoring of drug concentrations is warranted in children treated with preparations containing CBD, including medicinal cannabis [73].

The third study [74] explored the anticonvulsant effects and tolerability of plant derived-CBD in in vitro and in vivo rat models of seizures and tested CBD in combination with common AEDs. CBD significantly improved status epilepticus-like conditions; moreover, co-administration with AEDs was well tolerated and did not involve negative drug-drug interactions. Such favourable effects, obtained in a broad range of in vitro and in vivo seizure models, suggest that CBD should be further explored for application to a wide range of human epilepsies.

Finally, a multisite open-label study using the purified 98% oil-based CBD extract, has recently released data for 27 patients treated for more than 12 weeks [75]; about half the patients experienced a 50% reduction in seizures, and four patients were seizure-free. Sleepiness, fatigue, diarrhoea, and altered appetite were each reported by more than 10% of participants [75].

## 8 CLINICAL TRIALS OF CANNABIDIOL ADMINISTRATION IN EPILEPSY

Lennox-Gastaut syndrome (LGS) may be caused by several conditions, including brain malformations, severe head injury, CNS infection, and inherited degenerative or metabolic disorders; no cause can be found in 30-35% of cases. Onset is usually before 4 years of age. Patients commonly have frequent seizures of different types, including convulsive and atonic seizures. Drug resistance is a major feature of the syndrome. Most children with LGS display a degree of impaired intellectual functioning or information processing, development delay, and behavioural disturbances. It has been estimated that there are 14,000-18,500 *pochi?* patients with LGS in the US and 23,000-31,000 in Europe [76].

The purified 98% oil-based CBD extract described in the previous section was initially developed to treat two paediatric forms of epilepsy, DS and LGS. Several clinical trials have been launched to test the CBD extract in children with these forms of TRE [76].

### 8.1 Clinical Trials

**Phase 2**. A phase 2-3, double-blind, randomized, placebo-controlled, parallel group trial of the safety, tolerability, pharmacokinetics, and efficacy of the CBD extract was launched in October 2014, to test the adjunctive effect of a single CBD dose and of multiple CBD doses in children with DS receiving AEDs. It consisted of two parts: part 1 addressed pharmacokinetics and dose-finding aspects over a 3-week treatment period in children, whereas part 2 was a placebo-controlled safety and efficacy evaluation over a 14-week treatment period involving 80 children. The patients completing the study are now eligible to receive the agent under a long-term open-label extension study [76].

**Phase 3.** Phase 3 of the trial began in March 2015, after part 1 was completed in February 2015. The dosage was set at 20 mg/kg by a Data Safety Monitoring Committee after evaluation of the safety and pharmacokinetic data obtained in part 1 [76].

Part 2 involved 14-week administration of the CBD extract or placebo to 100 patients not involved in part 1, to assess CBD safety and efficacy as an adjunctive antiepileptic agent. The primary outcome measure was the percent change in the frequency of convulsive seizures during the maintenance phase compared with baseline and with the placebo group. Several efficacy and safety secondary outcome measures were included. Participants are now eligible to receive the agent under a long-term, open-label extension study [76].

The second, phase 3 clinical trial of the CBD extract began in April 2015, to assess its safety and efficacy as an adjunctive antiepileptic agent over a 14-week period. It involved three treatment arms - 10 mg/kg/day, 20 mg/kg/day, and placebo - each with 50 patients. The primary outcome measure was the percent change in convulsive seizure frequency in the maintenance phase both compared with baseline and between the CBD groups and the placebo group. Several efficacy and safety secondary outcome measures were also included. Participants are now eligible to receive the agent under a long-term, open-label extension study [76].

A phase 3 randomized, double-blind, clinical trial began in May 2015, to examine the dose-ranging safety and efficacy of the CBD extract as an adjunctive antiepileptic agent in LGS. It envisaged three treatment arms - 20 mg/kg/day and 10 mg/kg/day CBD extract and placebo - each involving 50 patients, and a 14-week titration period followed by a 12-week maintenance period. The primary outcome measure was the percent change both compared with baseline and between the treated groups and the placebo group. Several efficacy and safety secondary outcome measures were also included. Participants are now eligible to receive the agent under a long-term, open-label extension study [76].

Another phase 3 randomized, double-blind clinical trial of the CBD extract in LGS patients began in June 2015. The programme included two studies of the extract and involves a 2-week titration period followed by a 14-week maintenance period. One study comprised two arms, 20 mg/kg CBD and placebo, each involving 50 patients, whereas the second study also included a low-dose treatment arm (hence an additional group of 50 patients). The primary efficacy outcome measure in both studies was the percent change in the number of drop seizures both compared with baseline and between the CBD groups and the placebo patients. Several secondary efficacy and safety outcome measures were included. Patients are now eligible to receive the CBD extract under a long-term, open-label extension study [76].

## 9 CONCLUSIONS AND REMARKS

Epilepsy is a CNS disorder characterized by uncontrollable twitching of upper or lower limbs and/or seizures. It is estimated that standard medications fail to induce significant symptom alleviation in as many as 30 percent of patients.

The data reviewed herein demonstrate that cannabinoids provide neuroprotection against brain excitability. They seem to induce at least partial restoration of neurotransmitter dysfunction, inducing an anticonvulsant effect that may be the biological substrate of the complex neurochemical effects reported in experimental and clinical studies. A large body of data suggests that cannabinoids can be harnessed as antiepileptic agents [77-82].

However, in the absence of data from placebo-controlled clinical studies, reports of the ability of cannabis to ameliorate epileptic symptoms remain anecdotal [83]. Lately, CBD has begun to be used to mitigate intractable paediatric epilepsy by clinicians [84] as well as patients' parents [85,86]. Recently published observational data also support the anti-seizure effect of CBD in adolescents. A retrospective chart review of children and adolescents receiving oral cannabis extract at a Colorado epilepsy centre found reduced seizure frequency in up to 57% of patients as well as improved behaviour/alertness (33%), language (10%), and motor skills (10%) [87]. In Israel, a 2016 retrospective study of a multicentre cohort of 74 TRE patients found that administration of a CBD extract provided by two local licensed growers (standardized to possess a 20: 1 CBD to THC ratio) for at least 3 months induced seizure relief in 89% of children and improved behaviour/alertness, language, communication, motor skills, and sleep [88].

In late 2013, the US Food and Drug Administration granted orphan drug status to an imported, pharmaceutically standardized CBD extract (Epidiolex^®^, GW Pharmaceuticals) as an experimental treatment for paediatric epilepsy. Clinical trials of its safety and efficacy in children with severe forms of TRE, such as DS, began in 2014 [89]. A consortium of 10 epilepsy centres is currently collecting prospective data on children and young adults taking Epidiolex^®^ [90]. According to the preliminary results, presented in April 2015 to the 67^th^ Annual Meeting of the American Academy of Neurology, it reduced seizure frequency by 54% over a 12-week period [91]. Trial data, reported to the Annual Meeting of the American Epilepsy Society in late 2015, showed that adjunctive Epidiolex^®^ treatment afforded long-term seizure reduction in 40% of adolescent subjects [92]. Data from an open-label trial, published online in December 2015, also showed an almost 40% median reduction in seizures in adolescents receiving Epidiolex^®^, suggesting that CBD may provide seizure relief having an adequate safety profile in children and young adults with highly TRE [93]. According to another observational study, 70% of TRE children treated with Epidiolex^®^ and clobazam experienced a more than 50% reduction in seizure frequency, suggesting that CBD is a safe and effective treatment [94]. Clinical trials of Epidiolex^®^ and several state-sponsored trials using CBD extract are ongoing and are expected to confirm the encouraging results obtained in earlier, smaller trials.

Finally, among patients with the Dravet syndrome, cannabidiol resulted in a greater reduction in convulsive-seizure frequency than placebo and was associated with higher rates of adverse events [95] and it might reduce seizure frequency and might have an adequate safety profile in children and young adults with highly treatment-resistant epilepsy [96].

## Figures and Tables

**Table 1:** Antiepileptic drugs grouped by their main mechanism of action.

**Phenytoin, Carbamazepine**	**Block voltage-dependent sodium channels at high firing frequencies**
**Barbiturates**	**Prolong GABA-mediated chloride channel openings** **Some blockade of voltage-dependent sodium channels**
**Benzodiazepines**	**Increase frequency of GABA-mediated chloride channel openings**
**Felbamate **	**May block voltage-dependent sodium channels at high firing frequencies** **May modulate NMDA receptor via strychnine-insensitive glycine receptor**
**Gabapentin**	**Increases neuronal GABA concentration** **Enhances GABA mediated inhibition**
**Topiramate** • **Blocks voltage-dependent sodium channels at high firing frequencies** • **Increases frequency at which GABA opens Cl- channels (different site than benzodiazepines)** • **Antagonizes glutamate action at AMPA/kainate receptor subtype** • **Inhibition of carbonic anydrase**	
**Lamotrigine**	**Blocks voltage-dependent sodium channels at high firing frequencies** **May interfere with pathologic glutamate release**
**Ethosuximide**	**Blocks low threshold, “transient” (T-type) calcium channels in thalamic neurons**
**Valproate**	**May enhance GABA transmission in specific circuits** **Blocks voltage-dependent sodium channels**
**Vigabatrin**	**Irreversibly inhibits GABA-transaminase**
**Tiagabine**	**Interferes with GABA re-uptake**
**Levetiracetam**	**Binding of reversible saturable specific binding site** **Reduces high-voltsge- activated Ca^2+ ^ currents** **Reverses inhibition of GABA and glycine gated currents induced by negative allosteric modulators**
**Oxcarbazepine**	**Blocks voltage-dependent sodium channels at high firing frequencies** **Exerts effect on K+ channels**
**Zonisamide**	**Blocks voltage-dependent sodium channels and ** **T-type calcium channels**

**Table 2:** Experimental and human regarding the role of cannabidiol in different model of epilepsy.

**Compound**	**Type of animal model**	**Results**	**References**
Phytocannabinoid cannabidivarin (CBDV)	1) Epileptiform local field potentials (LFPs) 2) Maximal electroshock (MES) 3) Audiogenic seizures in mice 4) Pentylenetetrazole (PTZ) and pilocarpine-induced seizures in rats	CBDV is an effective anticonvulsant in a broad range of seizure models	[61]
Phytocannabinoid cannabidivarin (CBDV)	1) Audiogenic seizures in mice 2) Pentylenetetrazole (PTZ) and pilocarpine-induced seizures in rats	CBDV induces significant anticonvulsant effects in all three seizure models	[62]
Phytocannabinoid cannabidivarin (CBDV)	Pentylenetetrazole (PTZ)-induced seizures in rats	CBDV induces significant anticonvulsant effects on PTZ seizure	[63]
Phytocannabinoid cannabidivarin (CBDV)	Epileptiform neuronal spike activity in hippocampal brain slices exposed to a Mg(2+)-free solution	CBDV reduced epileptiform burst amplitude and duration	[64]
WIN55.212-2 and URB597	Amygdala kindling model of temporal lobe epilepsy	WIN55.212-2 significantly delayed the progression of seizure severity, whereas URB597 did not affect seizure development	[65]
**Human data**	Twelve patients with refractory mesial temporal lobe epilepsy due to hippocampal sclerosis received a [(18)F]MK-9470 scan to assess the in vivo availability type 1 cannabinoid (CB1) receptor	CB1 receptor availability showed opposite changes in different brain regions that are involved by complex partial seizures in refractory mesial temporal lobe epilepsy with hippocampal sclerosis	[66]
**Human data**	Comparative expression profiling and quantitative electron microscopic analysis of postmortem samples from subjects with no signs of neurological disorders (controls) and surgical specimens of epileptic hippocampal tissue removed from patients with intractable temporal lobe epilepsy	The impaired neuroprotective effects of endocannabinoids in human epileptic hippocampus suggest that downregulation of type 1 cannabinoid receptor and related molecular components of the endocannabinoid system may compound the harmful effects of network hyperexcitability	[67]
**Human data**	Measurement of endocannabinoid levels in cerebrospinal fluid (CSF) of drug-naive patients with temporal lobe epilepsy	Patients exhibited significantly reducted CSF anandamide levels compared with healthy controls	[68]
